# Non‐Destructively Quantifying the Whole‐Course Growth and Drug‐Response of PDOs by an Automatic Microfluidic System Utilizing Chemiluminescence Detection

**DOI:** 10.1002/advs.202512951

**Published:** 2025-10-24

**Authors:** Yu Zhang, Daoyun Wang, Zhicheng Huang, Nan Zhang, Zhina Wang, Xin Wu, Anlan Zhang, Runzhi Yang, Tong Li, Zhibo Zheng, Yuxiao Lin, Naixin Liang, Zewen Wei

**Affiliations:** ^1^ Department of Biomedical Engineering School of Medical Technology Beijing Institute of Technology Beijing 100081 China; ^2^ Department of Thoracic Surgery, Peking Union Medical College Hospital Chinese Academy of Medical Sciences and Peking Union Medical College Beijing 100730 China; ^3^ Department of Pulmonary and Critical Care Medicine 2 Emergency General Hospital Beijing 100028 China

**Keywords:** biosensor, continuous monitoring, microfluidic chip, patient‐derived organoids

## Abstract

Patient‐derived organoids (PDOs) have become promising tools in precision medicine research. While conventional imaging techniques provide morphological assessment, they fail to reveal crucial molecular‐level changes. Monitoring secreted biomarkers presents an alternative approach that can deliver real‐time physiological data throughout the growth and drug response process. In this study, the non‐destructive quantification for the whole‐course growth and drug‐response of PDOs is first realized using a multifunctional microfluidic chip‐based system that integrates culturing, drug incubation, and biomarker detection. To validate the feasibility of this method, Carcinoembryonic Antigen (CEA), a broad biomarker, is selected to investigate its correlation with both organoid growth (over 6 days) and drug response (over 72 h).  The stable culture of organoids within the device is enabled by the integrated system, with net CEA accumulation being continuously monitored to assess growth rate. Additionally, finer‐resolution drug response monitoring is achieved by measuring the same organoids at multiple intervals. The drug testing results demonstrated concordance with clinical outcomes in patients. Such continuous monitoring of biomarkers has the potential to effectively respond to the growth and drug‐response of the PDOs, with a fine‐grained interpretation of organoids being provided as a patient prognostic evaluation.

## Graphical abstract

The detection of secreted biomarkers enables molecular variations monitoring of organoid status. In this study, the non‐destructive quantification for the whole‐course growth and drug‐response of PDOs is first realized using a multifunctional microfluidic chip‐based system(ASMO) that integrates culturing, drug incubation, and biomarker detection. Results aligned with clinical outcomes, enabling non‐destructive prognostic evaluation.

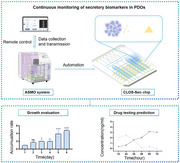

## 1 Introduction

Patient‐derived organoids (PDOs) are 3D in vitro models that recapitulate key histopathological, genetic, and phenotypic features of primary tumors.^[^ ^1^, ^2^ ^]^ Their high fidelity to primary tissues has positioned PDOs as promising platforms for disease modeling and drug sensitivity testing.^[^ ^3^, ^4^, ^5^ ^]^ Before being applied in disease research, PDOs usually undergo a complex and dynamic self‐assembly process that begins with cell aggregation, further differentiation, and ultimately the formation of a specific tissue structure.^[^ ^6^ ^]^ By closely observing this entire growth process, the growth trend of the organoids can be fully assessed.^[^ ^7^, ^8^ ^]^ Simultaneously, appropriate interventions (such as passaging^[^ ^9^ ^]^ and supplementation of specific factors^[^ ^10^, ^11^ ^]^) could be implemented promptly to promote proliferation or differentiation for better disease modeling. Apart from tracking the growth process, tracking the whole course of drug response of organoids is more important.^[^ ^12^, ^13^ ^]^ As drugs usually vary in speed and duration of action, detecting the drug responses at the wrong point in time can often lead to a misinterpretation of the drug's efficacy and, consequently, misguided treatment.^[^ ^14^, ^15^ ^]^ Since the growth and drug responses of organoids are time‐dependent, a detailed and accurate diagram can only be obtained by observing the whole course at finer time intervals.^[^ ^16^ ^]^

The exploration of continuous monitoring of organoids started from simply observing organoid profiles by microscopy, as the morphology was the most intuitive way to monitor cell status.^[^ ^17^, ^18^ ^]^ Several sophisticated microscopy techniques, including high‐content confocal^[^ ^19^ ^]^ and light‐sheet imaging,^[^ ^20^, ^21^ ^]^ were also applied to acquire 3D morphology with enhanced resolution. After the basic observation of organoids' morphology was realized, a requirement for detecting molecular variations of organoids to understand key biological processes and evaluate drug responses has been raised. The advent of dyes fulfilled this need by amplifying signals at the molecular level, allowing not only the determination of dead and alive, but also the characterization of specific proteins of organoids maturation markers. In the latest high‐content imaging technology, the target protein is labeled by using specific live cell dyes, enabling the tracking of organoid growth and drug sensitivity throughout the entire process.^[^ ^22^ ^]^ Apart from directly observing fluorescently stained organoids, detecting specific biomarkers secreted by organoids in culture medium is an attractive alternative to profile the growth status and molecular characteristics of organoids.^[^ ^23^, ^24^ ^]^ Such indirect detection eliminated the external interference on organoids, therefore maintaining the natural growth trend of the organoid. Meanwhile, measuring cumulative metabolite levels over time improves sensitivity for low‐abundance markers, avoiding the need for high transient concentrations required in single‐time‐point assays.

 Various methods, such as Enzyme‐linked immunosorbent assay (ELISA),^[^ ^25^, ^26^ ^]^ Electrochemistry,^[^ ^27^, ^28^ ^]^ Chemiluminescence,^[^ ^29^, ^30^ ^]^ Mass spectrometry,^[^ ^31^ ^]^ and Surface‐enhanced Raman spectroscopy^[^ ^32^ ^]^ have been developed to detect the content in culture medium. Among these methods, chemiluminescence is favored for real‐time detection of trace substances due to its stable performance and fast detection. However, in common detection scenarios, the test substance would be removed from the original system to the detecting devices.^[^ ^23^, ^33^ ^]^ The process of movement, which usually involves either direct pipetting or enrichment by centrifugation, will inevitably result in a programmed loss of the target analyte. This presents a critical challenge for low‐abundance biomarkers (pg∼ng/mL) secreted by limited cellular populations (10 000–100 000 cells^[^ ^7^, ^34^ ^]^), where such losses disproportionately compromise detection fidelity. Hence, methods that can be integrated in situ into culture systems with high sensitivity and low detection limits are yet to be realized.

To achieve the non‐destructive whole‐course monitoring of PDOs during the growth and drug response, the Chemiluminescent‐based organoids Statue Sensor (CLOS‐Sen) was developed, which enabled in situ marker detection over multiple time intervals to predict the growth trend and drug response. Moreover, based on this chip, an Automated System for Whole‐Course Monitoring of Organoids (ASMO) was developed to manipulate the operation of the chip and to realize the target marker detection within 2 h. In detail, in situ detection units were integrated with microfluidic channels to enrich biomarkers without fluid transfer losses; time‐partitioned chambers were employed to prevent cross‐contamination across sampling intervals; automated perfusion was conducted to maintain the nutrient supply while driving the sensor generation and actuation. In conclusion, for the first time, a non‐destructive whole‐course monitoring of PDOs was achieved by continuously detecting the biomarkers in situ. This achievement provides a continuous analyze method for the long‐term tracking of organoids status and facilitates precise evaluation of patient prognosis through comprehensive organoids drug response profiling.

## 2 Results and Discussion

### 2.1 The Automated System for Whole‐Course Monitoring of Organoids (ASMO)

**Figure 1:**
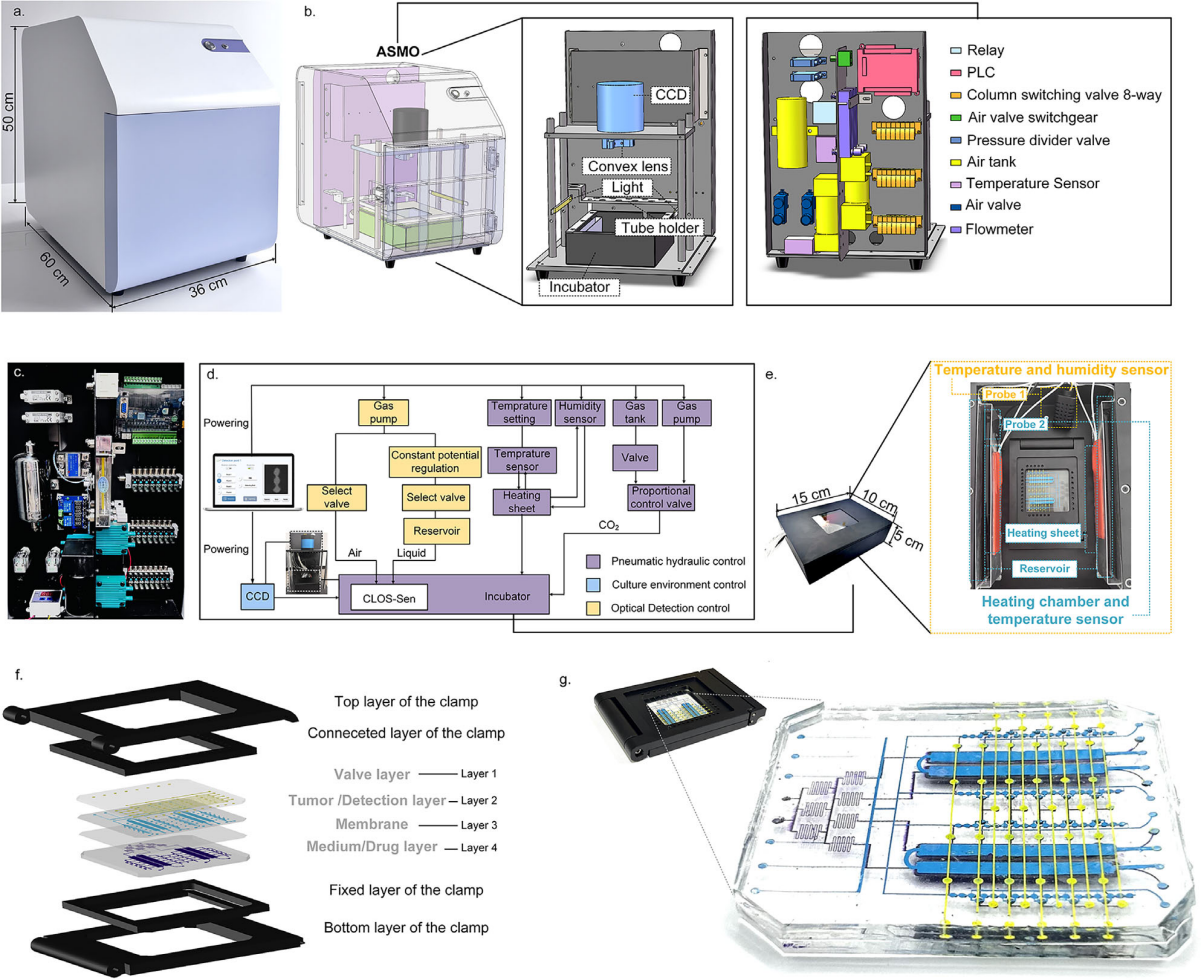
The Automated System for Whole‐Course Monitoring of Organoids (ASMO). a) The photo of the ASMO. b) The perspective view of the ASMO. The entire instrument was divided into two compartments by a backplane: the culture and detection compartment in front of the backplane, and the control compartment behind it. c) The physical assembly drawing of the control compartment. d) The schematic of the ASMO workflow. e) The incubator was designed to be capable of providing stable temperature and humidity. The probe 1 corresponding to the temperature and humidity module was used to react the internal environment of the incubator. The probe 2 corresponding to the temperature sensing module was used to control the temperature of the heating chamber. f) The exploded view of the clamps and CLOS‐Sen chip. g) The photo of the clamps and CLOS‐Sen chip.

During organoids' growth and drug response, continuous observation is essential for capturing dynamic changes. While traditional bright‐field microscopy tracks morphological changes, it fails to detect molecular‐level variations. To precisely monitor whole‐course development of organoids without cell destruction, a new method has been proposed, from focusing on the cell morphology to studying its biomarkers. For the realization of this method, an Automated System for Whole‐Course Monitoring of Organoids (ASMO) was developed to monitor the key secreted biomarker during the growth and drug response of organoids. **Figure** **1**a shows the physical view of the ASMO (36 cm in length, 60 cm in width, and 50 cm in height). As depicted in Figure 1b, the instrument was divided into front and rear compartments by a backplane. The front chamber housed the incubator and CCD detector, which together with the outer enclosure and backplane form a fully light‐sealed darkroom during detection. The rear compartment integrated all control components for easy maintenance and operation. Components were equipped compactly on the backplane according to the layout design to minimize the overall instrument footprint (shown in Figure 1c).

The logic diagram of the ASMO was shown in Figure 1d. In terms of functional modules, the whole system consisted of a pneumatic hydraulic control module, the culture environment control, and the optical detection control module. A pre‐programmed sequence of commands was received from the external PC and transmitted to the central circuit board of the control module. Under the guidance of commands, tasks were sequentially executed in separate functional areas of the Chemiluminescent‐based organoids Statue Sensor (CLOS‐Sen). The fluorescence data generated by the chemiluminescent sensor were read by a CCD, further processed by ImageJ to quantify the intensity of fluorescence. For continuous observation over a long period of time, the CLOS‐Sen chip was equipped in an incubator (15 cm in length, 10 cm in width, and 5 cm in height), which facilitated the maintenance of an overall stable environment. As shown in Figure 1e, the incubator consisted of two heating sheets, two reservoirs, one temperature sensor, and one temperature and humidity sensor. The temperature and humidity were supplied through the heated water in the reservoirs. The temperature sensor connected to the heating sheets was set to start heating at 41 °C and stop heating at 42 °C (detected by probe 2). With this setting, the temperature and humidity (detected by probe 1) in the incubator can be maintained at a constant temperature of 37–38 °C and a cross‐humidity of 90∼95% (shown in Figure S1, Supporting Information).

CLOS‐Sen chip, the core component of the system, was composed of four layers: the valve layer, the tumor/detection layer, the membrane layer, and the culture/drug layer. Due to the high biocompatibility, hydrophobicity, transparency, and breathability, the polydimethylsiloxane (PDMS) can serve both as a chamber for organoid culture and as a substrate for sensor generation. Conventional pinhole‐based insertion and removal introduce gas when changing fluids, causing disturbance to the cultivation environment. To facilitate easy installation and stability of the environment for long‐term cultivation, a delicate fixture (shown in Figure 1f)was designed to fix the inlet and outlet of the chip. As depicted in Figure 1g, the chip was layered with colors to show its overall design (yellow in layer 1, blue in layer 2, transparent in layer 3, and purple in layer 4).

**Figure 2:**
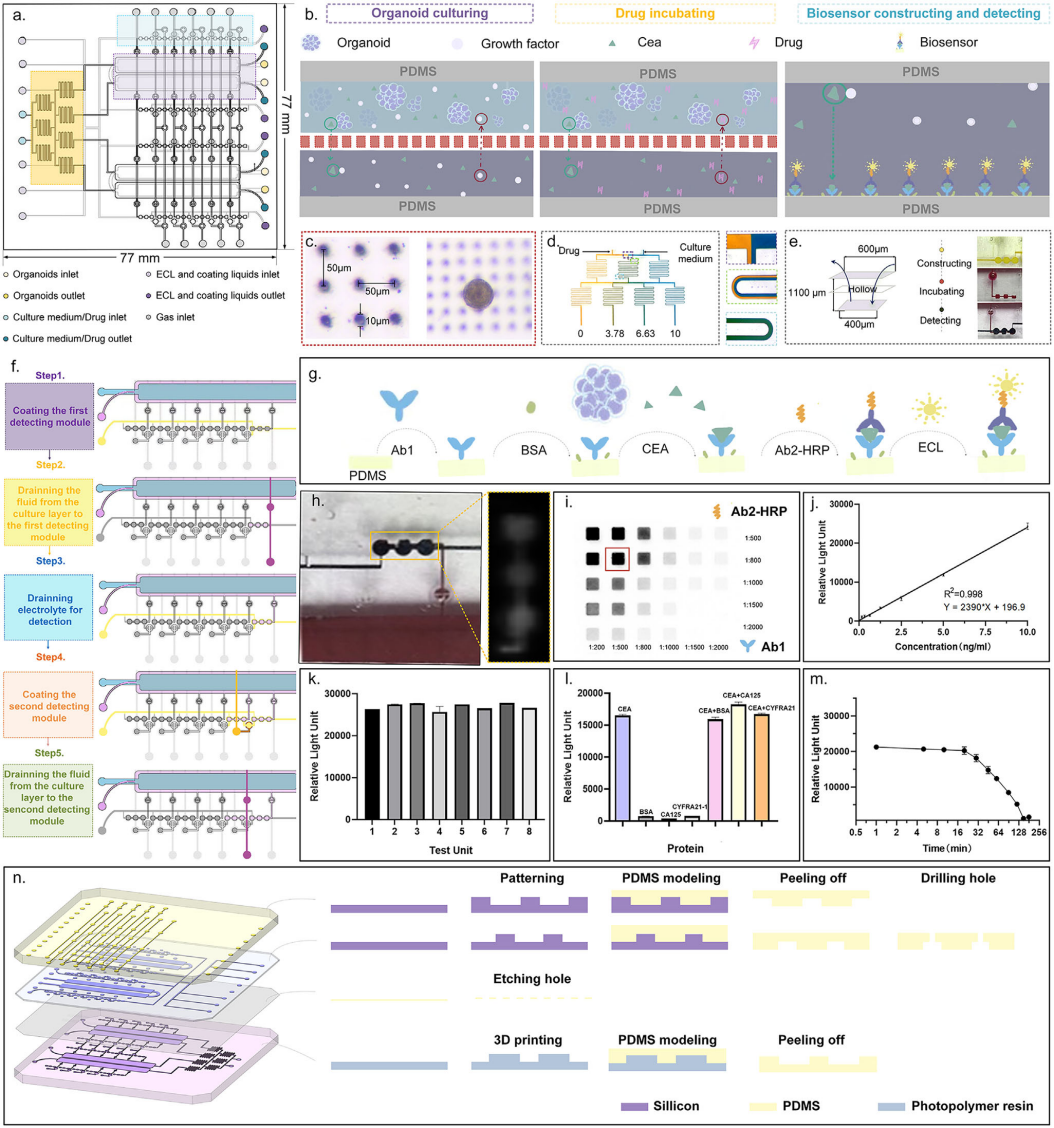
Design concept and working principle of the CLOS‐Sen chip. a) The vertical view of the CLOS‐Sen chip. The CLOS‐Sen chip involved three functional areas: the organoids culturing area, drug incubating area, and biosensor constructing area. b) Design concept and functional components of three functional areas. c) The physical image of a porous membrane used to supply nutrients for organoids culture. d) The physical image of the drug concentration generator. e) The liquid flow direction of the sensor unit. f) Process flow for biosensor construction and biomarker detection. Grey indicated closed air valves or inactive flow channels with no liquid movement, while colored sections represented open valves and active liquid flow within the system. g) The construction scheme of a chemiluminescent biosensor for CEA detection. h) The detection of CEA by capturing the chemiluminescent photo. i) The optimization of the concentration about Ab2‐HRP and Ab1. j) The standard curve of CEA detection using a chemiluminescent biosensor. k) The repeatability of the biosensor in the presence of 10 ng mL^−1^ standard CEA. (n = 3) l) The selectivity of the biosensor in the presence of different 5 ng mL^−1^ interferences. (n = 3) m) Relationship between sensor luminous intensity and detection time point. Luminous intensity detection was stabilized within 15 min. n) Different machining processes for the four‐layer structure of the chip.

**Figure** **2**a presents the vertical view of the CLOS‐Sen chip (77 mm in width, 77 mm in length). Three functional modules (organoid culture, drug incubation, and biosensor‐based detection) were integrated on one chip. In order to make it easier to expand the throughput of detection units on the chip, the functional areas were designed to be symmetrical and independent. Figure 2b depicts the schematic diagram of the chip partitioning to realize the three functional modules. The detailed parameters of the key chip components are shown in Figure S2 (Supporting Information). To enrich organoids' secretions while maintaining nutrient growth, the heights of the flow channel in the tumor and medium/drug layer were designed to be 200 and 800 µm. The membrane sandwiched between these two layers was designed to be 100 µm, which was not only flexible enough to enable the valve to work, but also a barrier layer to prevent interference with upward and downward fluid flow (verified in Figure S3, Supporting Information). As shown in Figure 2c, the laser‐etched micropores on the membrane were 10 µm in size and 50 µm in spacing to realize the exchange of nutrients and secreted substances. To introduce drug interventions, a gradient generator was connected to the drug layer to generate uniform drug concentrations. This design employed a minimal Christmas‐tree microfluidic structure to generate four concentration gradients (0, 3.78, 6.63, and 10 µm, as simulated in Figure S4, Supporting Information by COMSOL). Figure 2d demonstrated the uniform mixing effect of this flow channel structure at a flow rate of 5 µL min^−1^. Expansion of drug concentration intervals can be conveniently accomplished by adding tree branches to the structure.

Commonly used protein detection methods often require complex modification processes and bulky detection instruments that are difficult to integrate on microfluidic chips. Due to the advancement in microfabrication, electrochemical methods are considered to be the most likely means of real‐time monitoring for integration on the chip. However, the electrode lifetime and data reproducibility are bottlenecks for its application. Based on the hydrophobic interaction of the PDMS surface, a chemiluminescent sensor was found to be generated in situ upon the weak intermolecular forces between the PDMS substrate and protein.^[^ ^35^, ^36^ ^]^ This in situ integration approach significantly reduces fabrication complexity while maintaining sensitivity adequate for detection requirements.

As shown in Figure S5 (Supporting Information), the protein (Carcinoembryonic Antigen, CEA used in this article) was proven to be bound to the surface of PDMS successfully by a fluorescent labeling assay. In order to meet the multiple time points detection, an array of six time points detection was designed based on three repeating units (shown in Figure 2e), with a dimension of 400 µm *400 µm* 1.1 mm. Figure 2f illustrates the detailed execution flow for sensor generation and detection, which were accomplished sequentially by precise valve control. Valve type 1 (Figure S6a, Supporting Information) and type 2 (Figure S6b, Supporting Information) were programmed to separately control the targeted medium liquid diversion layer and the on/off control of each detection unit. Through the coordinated operation of two valves, only the medium under test entered the detection channel during each cycle, thereby achieving fluidic isolation between units. In detail, 5× Ab1 and 1× blocking solution were sequentially introduced into the sensor‐generated flow channel at 0 h. The solutions were incubated for 20 min, and then excess liquid was washed away with PBS. At 24 h, the medium in the incubation chamber was introduced into the detection chamber by turning on the valves between these two chambers. To minimize the impact of metabolites, the culture medium in the incubation chamber was replaced at each detection time point. Therefore, the measured analyte concentrations in the detection chamber represent 24‐h cumulative levels. After a 20‐min incubation of the medium, PBS was passed through to wash the chamber. Ab2‐HRP liquid was then introduced for another 20‐min incubation. At last, the detection liquid was introduced, and the detection was completed within 15 min. The entire chemiluminescent sensor generation mechanism was shown in Figure 2g. By detecting the fluorescence intensity of the detection units (shown in Figure 2h), the concentration of a specific factor was quantified according to the standard product. The concentrations of Ab1 (1:500) and Ab2‐HRP (1:800) were chosen for subsequent research through the orthogonal experimental design in Figure 2i. According to Figure 2j, the final sensor standard curve generated with the optimal parameters presented a high correlation coefficient of 0.998 and a minimum detection limit of 0.0242 ng mL^−1^. To validate the accuracy of the method, three standards and three actual culture media samples (post‐organoid culture) were analyzed using both ELISA and ASMO assays. As shown in Table S1 (Supporting Information), no statistically significant differences were observed between the results obtained from the two detection methods. Figure 2k showed that the sensor possesses repeatability when analyzing identical concentrations at varying locations. Its selectivity for CEA was also proven to be distinguished from other substances, including BSA, CYFRA21‐1, and CA125, as illustrated in Figure 2l. Notably, due to the inherent instability of the chemiluminescent substrate, accurate detection should be completed within 15 min (as shown in Figure 2m).

In order to achieve the processing required for the different channel heights, the layers were processed using different processes: photolithography was used to make the molds for the lower runner height layers (layer 1 and layer 2); 3D printing was used to make the molds for the higher channel layer (layer 4); and the membrane layers were commercially purchased membranes that were subsequently etched out. The detailed processing of each layer of the chip was shown in Figure 2n.

### 2.2 Whole‐Course Monitoring of Organoids Using CEA as an Indicator

CEA, a tumor‐associated glycoprotein, exhibits elevated serum levels in solid tumor patients, making it a valuable biomarker for early detection.^[^ ^37^ ^]^ So far, no practical studies of the real‐time secretion of organoids CEA have been performed. Based on our platform described above, the CEA secreted by the organoids can be detected continuously during growth and the drug response process. According to the correlation between CEA and tumor development at the preclinical and cellular levels,^[^ ^38^, ^39^ ^]^ a hypothesis has been proposed that CEA accumulation will increase when the tumor growth rate is rapid. To test this conjecture, image tracking, a commonly used real‐time monitoring tool, was used as the standard evaluation for the growth trend of organoids (shown in Figure S7a, Supporting Information). Supplemented staining for proliferator proteins (Ki67) of the organoids was applied to verify their proliferative capacity.

**Figure 3:**
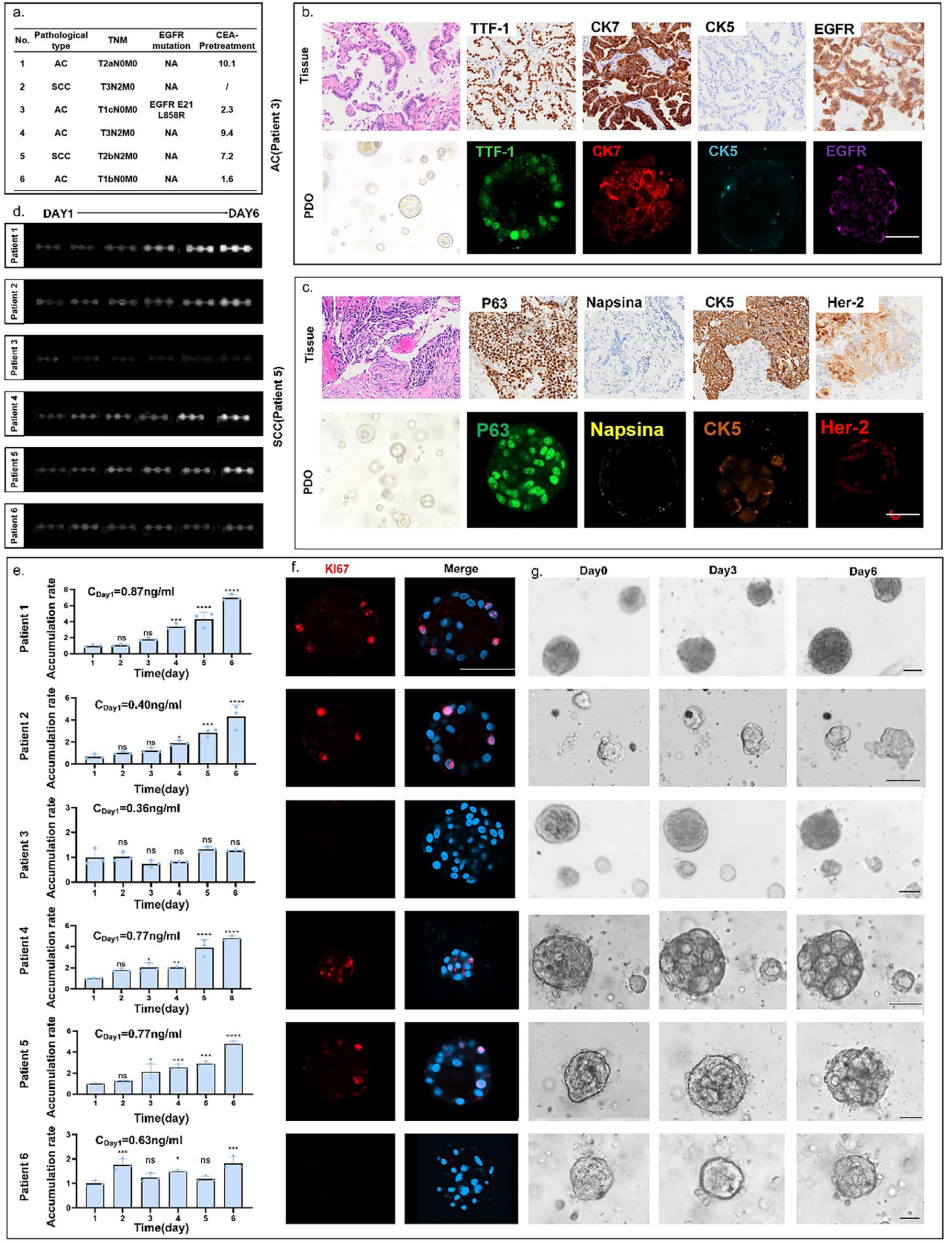
Real‐time monitoring of CEA concentration during organoid growth based on ASMO. a) A brief table of patient information. b) Up line: H&E and immunohistochemistry of primary tissue from lung adenocarcinoma cancer patient; down line: immunofluorescence of PDOs from lung adenocarcinoma cancer patient. c) Up line: H&E and immunohistochemistry of primary tissue from a lung squamous cell carcinoma patient; down line: immunofluorescence of PDOs from a lung squamous cell carcinoma patient. d) Photographs of net CEA accumulation detected in the organoid lines derived from patient 1–6. The daily CEA accumulation rate was compared with that of the first day; ^*^ *P* < 0.05, ^****^ *P* < 0.0001, ns: not significant. e) The quantitation of net CEA accumulation from six PDO lines on six consecutive days. f) Staining‐based assessment of organoids' proliferation status on Day 6. Scale bar: 50 µm. g) The representative charts of organoids derived from six patients on Day 0, 3, 6. Scale bar: 50 µm.

**Figure** **3**a presents 6 patients' brief pathological information (detailed pathological information was supplemented in Table S2, Supporting Information). By comparing the immunofluorescence of organoids with clinical H&E and immunohistochemical results of pathological tissues, the high fidelity of PDOs was confirmed to primary tissues (Figure 3b,c). After it was confirmed that the cultured PDOs retained the characteristics of the tumors, the secreted CEA concentration in six patients was detected using the ASMO system. By maintaining standardized culture conditions (e.g., consistent matrix lot, medium formulation, and temperature) across all PDOs, variability in CEA detection was minimized. As shown in Figure 3d, the chemiluminescence images from each detection unit reflect the net CEA accumulation of PDOs from 6 patients in 6 days with 24‐h intervals. In order to visually compare growth rates among the PDOs, the accumulation rate of CEA was defined, as depicted in Figure 3e. The CEA concentration was proven to be changed corresponding to growth states that were detected in PDOs of various subtypes and molecular classifications. In detail, the CEA concentration in PDOs from patients 1, 2, 4, 5 exhibited an increased accumulation rate, which indicated a faster growth capacity of organoids. This was consistent with their identification by staining for proliferation (Figure 3f) and picture tracking of growth trends (Figure 3g). The CEA concentration in PDOs from patients 3 and 6 remained stable in accumulation rate and low in overall secretion levels over the six days of testing. It suggested that the organoids from patients 3 and 6 were not in a growth spurt. Staining revealed no proliferation, and time‐lapse imaging demonstrated morphological atrophy as well. Since imaging typically allows only layer‐specific focusing for continuous tracking, this necessitates either compressing the overall culture volume for observation or sacrificing tracking of partial organoids. In contrast, secreted protein‐based detection better reflects the holistic status by simultaneously monitoring all organoids within the chamber.

According to clinical research, the reduction of CEA levels after drug action is a universal prognostic indicator.^[^ ^40^ ^]^ Thus, another hypothesis was proposed that drug stimulation alters CEA secretion patterns in organoids. With the CEA assay, an accurate temporal dimension of the organoids' response to the drug can be realized so as to provide a more comprehensive explanation of the drug's course of action. To verify the effect of drug stimulation, the organoids were under live‐dead staining and ATP assays on the last day.

**Figure 4:**
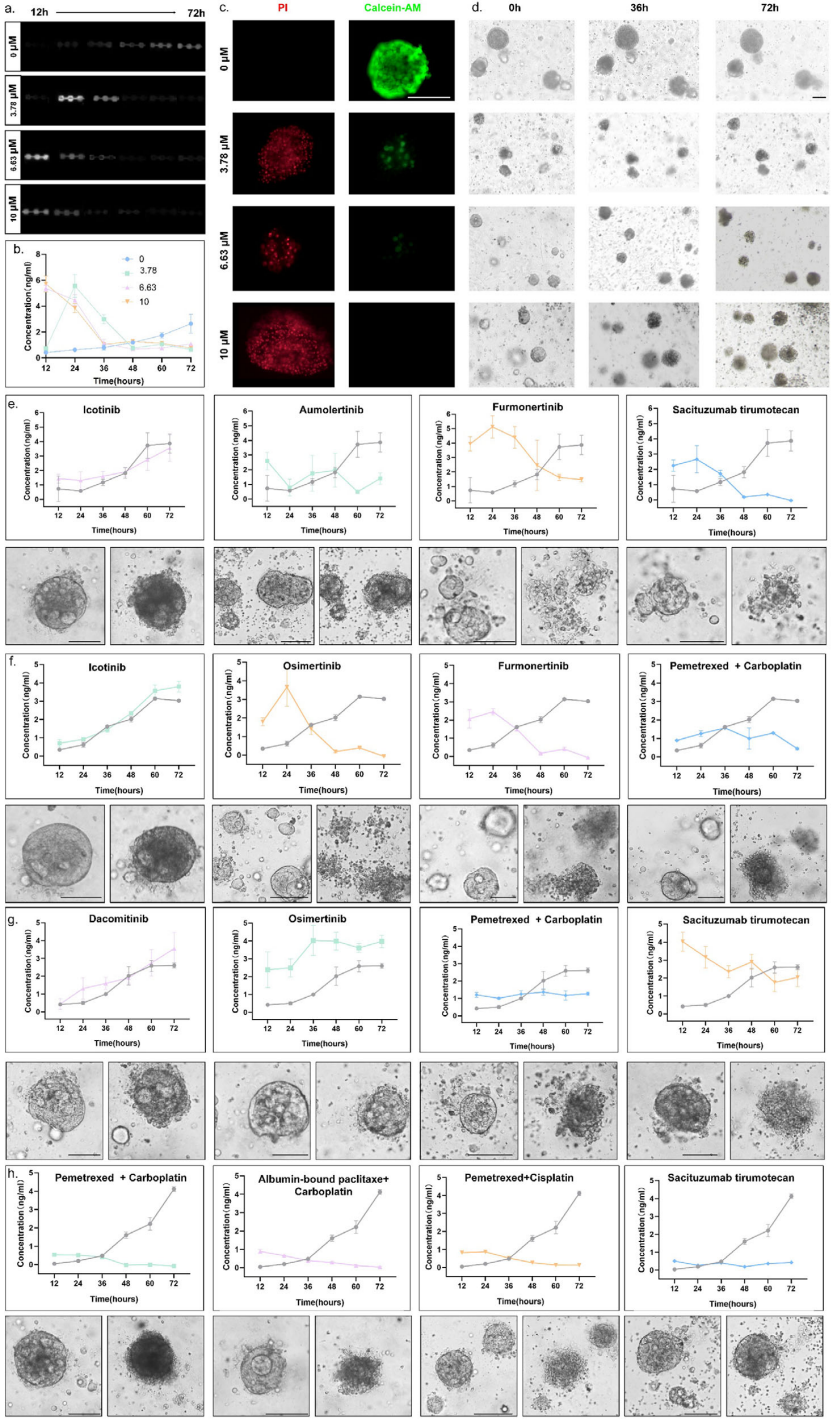
Real‐time monitoring of CEA concentration during organoids drug testing based on ASMO. a) Photographs of net CEA accumulation detected in the organoid lines derived from patient 3 under different drug concentrations (0, 3.78, 6.63, 10 µm). b) The quantitation of net CEA accumulation detected in the PDOs under different drug concentrations. The value for each test point was derived from three repeated tests. c) Staining‐based assessment of organoids' viability at 72 h. Scale bar: 50 µm. d) The representative charts of organoids under different drug concentrations on 0, 36, 72 h. Scale bar: 50 µm.e–h) Upline: the secretion levels of CEA under four clinical guidance drug tests in organoids derived from patients 7,8,9,10. The gray line represented the control group, and the colored line represents the experimental group (n = 10 µm). The value for each test point was derived from three repeated tests; downline: Images of organoids derived from patients 7,8,9,10 on the first day (left column) and the third day (right column) of drug incubation.

**Figure** **4**a portrays chemiluminescence images from each detection unit, reflecting net CEA accumulation of PDOs from Patient 3 at different drug concentrations of the organoids (0, 3.78, 6.73, 10 µm) in 72 h with 12‐h intervals. The EGFR mutation, identified by genetic testing (Figure S3, Supporting Information) and confirmed in patient‐derived organoid models (Figure S7b, Supporting Information), led to the selection of Osimertinib for subsequent drug testing. Based on the quantification of CEA concentration (Figure 4b), it was possible to find that the time point of peak CEA release from organoids was influenced by drug concentration. High drug concentrations (6.73 and 10 µm) triggered a surge of CEA release from the organoids, which subsided as the organoids died, ultimately lowering the total CEA accumulation. At low drug concentrations (3.78 µm), CEA release displayed fluctuations, as shown by an initial decrease, followed by a transient surge and a subsequent decrease. This fluctuation can be explained mechanistically by three successive phases.1) Early functional suppression – Sublethal drug exposure transiently inhibits cellular metabolic activity, reducing organoids secretory capacity; 2) Death‐mediated biomarker liberation – Subsequent drug‐triggered cell death pathways (apoptosis/proptosis) induce plasma membrane permeabilization, releasing intracellular CEA stores; 3) Secretory exhaustion – Depletion of viable cells terminates sustained CEA production, mirroring clinical observations where post‐treatment biomarker decline correlates with tumor necrotic burden.

As shown in Figure 4c, Calcein‐AM/PI staining of organoids on the last day demonstrates that higher concentrations of the drug have a stronger killing effect. It was also observed in the image tracing that both low and high drug‐concentrated organoids were killed compared to normal organoids (as reflected by cell crumpling and cell cluster cleavage in Figure 4d). Further, based on the conventional endpoint assay of ATP (Figure S7c, Supporting Information), the drug response of the organoids was measured on day 1 and day 3. At these two different time points, it was clear that the 3.78 µm concentrations did not perform an apparent kill on day 1, whereas they showed a significant kill on day 3. This warns that too early a time point detection will bring misjudgment of the results. Furthermore, due to the destructive lysing method, it was not possible to measure the same organoids again when missing the right time point. For the method of detecting secretions, the monitoring of the same type of organ can be continuous because of its non‐destructive nature. CEA‐based monitoring was shown to accurately reflect organoids' responses to drug stimulation. A high drug concentration directly induced a substantial release of CEA, whereas a low concentration delayed the time to peak CEA release.

After a comprehensive interpretation method for organoid‐based drug testing was established, this CEA‐based detection was then applied to evaluate organoids derived from patients who commonly require precise medication selection in clinical practice. As shown in Table S3 (Supporting Information), this cohort included individuals receiving third‐line therapy, adjuvant therapy and neoadjuvant therapy. These assays greatly enhanced our confidence on the accuracy and adaptability of the ASMO system. All drug candidates were selected from the NCCN Clinical Practice Guidelines in Oncology (Non‐Small Cell Lung Cancer, Version 4.2024). Figure 4e–g illustrates the drug responses of organoids from four patients. For each patient, the four optimal drug regimens selected based on the physician were tested at a uniform concentration of 10 µm, with a control group treated by medium containing 0.1% DMSO. The trend in CEA levels within 72 h for each drug was compared with that of the control group to evaluate the drug efficacy. Additionally, photographs of PDOs taken on Day 0 and Day 3 were used to observe the morphological changes of the PDOs' response to drug stimulation. In detail:

Figure 4e (Patient 7): This patient was in the advanced stage of the disease and was in the multi‐drug resistance phase, having previously been treated with and developed resistance to Icotinib (first‐generation EGFR‐TKI) and Aumolertinib (third‐generation EGFR‐TKI). To verify the drug resistance phenomenon and the accuracy of the ASMO system, Icotinib and Aumolertinib were tested on PDO. To compare the effect of alternative third‐generation EGFR‐TKI and ADC, which are the most competitive candidates for third‐line therapy (according to the NCCN guideline), Furmonertinib and Sacituzumab tirumotecan were also tested. The results exhibited that both Icotinib and Aumolertinib end up in the growth of tumors, which matched the previous multi‐drug resistance history. More importantly, Sacituzumab tirumotecan showed better tumor inhibition than Furmonertinib did, providing the physician with clear evidence for selecting Sacituzumab tirumotecan. Currently, Patient 7 received 2 courses of Sacituzumab tirumotecan and showed no tumor progression.

Figure 4f (Patient 8): This patient was in a moderate‐to‐advanced stage of the disease and had undergone surgical resection, with EGFR E19DEL mutation. According to the NCCN guideline, the first‐generation TKI (Icotinib), third‐generation TKIs (Osimertinib and Furmonertinib), and chemotherapeutic drugs (Pemetrexed + Carboplatin) were considered as candidates and tested by ASMO. The results showed that Icotinib was ineffective, while Osimertinib and Furmonertinib showed similar tumor inhibition, which is slightly better than Pemetrexed + Carboplatin did. Considering that adjuvant Osimertinib has demonstrated reliable safety and favorable efficacy in preventing recurrence and metastasis in Phase III clinical trials (ADAURA^[^ ^41^ ^]^), and has been approved as a reimbursable indication under the national health insurance system, the patient ultimately opted for postoperative adjuvant Osimertinib therapy. Currently, the patient wais in the postoperative adjuvant drug treatment phase, with no progression observed so far.

Figure 4g (Patient 9): This patient was a moderate‐to‐advanced patient with complex drug resistance, with EGFR L858R mutation and MET amplification. During previous treatment, the patient has developed resistance to multiple generations of TKIs and their combinations, including Dacomitinib, Furmonertinib, Osimertinib + Savolitinib, and Glumetinib. Currently, according to the NCCN guidelines, the only options are chemotherapy and ADC. Hence, Dacomitinib and Osimertinib were tested to verify the accuracy of ASMO, while the Pemetrexed+ Carboplatin and Sacituzumab tirumotecan were tested to help the physician select drugs. The results showed that Sacituzumab tirumotecan exhibited much better tumor inhibition than chemotherapy did. The patient was currently receiving the treatment of Sacituzumab tirumotecan and showed no progression.

Figure 4h (Patient 10): This patient was a patient with moderate‐to‐advanced disease, with no EGFR mutation. The neoadjuvant therapy was considered before surgery. According to the NCCN guideline, the treatment option included multiple chemotherapy regimens (Pemetrexed + Carboplatin, Pemetrexed+ Albumin‐bound paclitaxel, Pemetrexed+ Cisplatin). Sacituzumab tirumotecan was also tested. The results showed that all chemotherapy regimens showed good tumor inhibition. Considering the patient's tolerance level, Pemetrexed+ Carboplatin was selected. It is worth mentioning that during the period of ASMO testing, the CheckMate 816 trial^[^ ^42^ ^]^ provided conclusive evidence that PD‐1 inhibitor plus chemotherapy significantly improved the OS. Therefore, Pemetrexed+ Carboplatin+ Pembrolizumab was selected for the patient. The patient was currently in the neoadjuvant treatment phase and has achieved a partial response (PR) according to RECIST 1.1 criteria.

Overall, the broad applicability of CEA as a functional biomarker in lung cancer organoids was confirmed by the CEA detection using the ASMO system. The CEA secretion trend of the organoids was shown to be correlated with the growth trend in PDOs of various subtypes and molecular classifications. Furthermore, under various clinical drug treatments, cellular responses to drug stimulation were reliably reflected by CEA dynamics in PDOs. Compared to conventional single‐endpoint assays, a more detailed temporal analysis of drug effects was enabled by the developed approach. Most importantly, predictions of drug efficacy based on CEA trends in organoids were consistently aligned with the actual clinical outcomes of the patients. We must acknowledge that we have only established preliminary hypotheses and validation regarding the relationship of CEA secretion in tumor organoids. Further exploration of the potential mechanisms influencing it is still required to realize a more systematic analysis for the future standardization of biomarker‐based PDO studies. Besides, CEA expressions were also observed and clinically validated in many other kinds of cancer, including colorectal cancer,^[^ ^43^ ^]^ gastric cancer,^[^ ^44^ ^]^ pancreatic cancer,^[^ ^45^ ^]^ and breast cancer.^[^ ^46^ ^]^ Therefore, for those cancers with CEA secretion, it was theoretically feasible to apply the ASMO system to the above kinds of tumors. For cancers or individual cases lacking CEA secretion, since the ability to detect secreted markers was the foundation of our platform, our platform retains fundamental adaptability to detect other characteristic or validated alternative biomarkers.^[^ ^47^ ^]^

## 3 Conclusion

This study developed an automated microfluidic platform capable of supporting long‐term culture, drug testing, and real‐time biomarker monitoring of PDOs, offering a method that enabled continuously detecting of biomarkers. By integrating miniaturized culture chambers with in situ biosensing arrays, the system enables continuous, non‐invasive monitoring of tumor biomarkers (e.g., CEA) at single‐cluster resolution (40∼50 organoids) over multiple time points.  This method offered superior temporal resolution compared to ATP assays for tracking dynamic drug responses and reduced sample consumption to one percent volume of ELISA methods while doubling processing speed without compromising accuracy. As organoid culture is a long‐term process (15–30 days), rapid cell expansion in later stages may cause biomarker secretion levels to exceed the detector's measurement range. This limitation can be overcome by optimizing the overall sensor performance, such as introducing nanomaterials or depositing metallic materials with high adsorption capacity, thereby extending the detection range. The system not only establishes a novel strategy for low‐abundance analytes detection but also advances lab‐on‐chip technologies through miniaturization and intelligent design, forming the basis for personalized treatment guidance. Future development of multi‐tumor‐marker monitoring systems could enable more comprehensive evaluation of organoid growth status.

## 4 Experimental Section

### Chip Design and Fabrication

The four‐layer microfluidic chip was fabricated from polydimethylsiloxane (PDMS; Sylgard 184, Dow Corning, USA) material. Each layer within the chip has different characteristics and manufacturing processes, which are detailed as follows: air Valve and Tumor Layers both featured channels with a height of 200 µm, fabricated through replica molding using two distinctively patterned master wafers; the culture layer was designed with an 800 µm height to ensure adequate nutrient supply for organoids maintenance. This layer was formed by replica molding using 3D printing technology. For the membrane layer, PDMS with a thickness of 100 µm (Zhong Ke, China) was implemented. To facilitate substance exchange, femtosecond laser systems (Coherent, USA) were employed to create uniformly distributed micropores in this PDMS membrane.

The replica of both types of molds was used by pouring the PDMS prepolymer solution onto the silicon wafer master mold/ photopolymer resin mold to obtain 2 mm‐thick PDMS pieces with channel structures for each layer. After the PDMS pieces for each layer were obtained through the above processes, they were cured at 80 °C for 20 min. Once cured, they were cut to the size of the chip. Ports were then pierced using a biopsy punch (1 mm diameter) to provide access to the chips for both cell injection and medium perfusion. Then, chips were assembled in three consecutive bonding steps: (i) air layer to tumor layer, (ii) tumor layer to the membrane layer, and (iii)membrane to medium layer. In all steps, bonding was achieved by oxygen plasma activation (1 min, 40 kHz, 200 W). Bonded parts were baked at 80 °C for at least 4h after each bonding step, and overnight after the entire chip was assembled.

### Organoid Culture

Fresh patient‐derived tissues were transported in DMEM/F‐12‐based organoids preservation buffer supplemented with 1% (v/v) penicillin‐streptomycin, 1% (v/v) HEPES, and 1% (v/v) GlutaMAX (Gibco, USA), maintaining cold‐chain integrity within 8 h post‐resection. Tissue processing commenced with mechanical dissociation into 1 mm^3^ fragments using sterile micro‐scissors under laminar flow, followed by enzymatic digestion in a PBS‐based solution containing collagenase type II (1000 U mL^−1^), dispase type II (2.4 U mL^−1^), primocin (100 µg mL^−1^), and the Rho kinase inhibitor Y‐27632 (10 µm) at 37 °C for 30–40 min. Enzymatic activity was quenched by adding 2 mL fetal bovine serum (FBS, Gibco, USA). The cell suspension underwent sequential filtration (100 µm nylon mesh) and red blood cell lysis buffer (Thermo Fisher, USA) before centrifugation (400 ×g, 5 min, RT). Pelleted cells were resuspended in Matrigel at a 1:2 (v/v) cell‐to‐Matrigel ratio and cultivated in the Petri dishes with medium. For growth measurement, the organoids were pre‐cultured in Petri dishes for three days before being transferred to the chip. For drug testing, the organoids were pre‐cultured in Petri dishes for ten days before being transferred to the chip. Prior to transfer, organoids were immersed with TE buffer at 4 °C for 15 min to liquefy the Matrigel, followed by centrifugation at 300 g. The organoids at the bottom were then collected and resuspended in Matrigel. By adjusting the ratio of organoids to matrix gel, the number of organoids (cell clusters larger than 30 µm were defined as organoids) was controlled to 150–200 per 100 µL to control the initial organoid count in each chamber. Then the aliquots were then precisely dispensed into microfluidic chambers via calibrated micropipette. Medium was added to the inlets and outlets of the chip to create a liquid seal. After incubating at 37 °C for 20 min, the chip was subsequently perfused with organoids medium formulated as: Advanced DMEM/F12 (Gibco, USA) supplemented with 50 ng mL^−1^ Noggin, 100 ng mL^−1^ R‐spondin 1, 1× B27, 10 mm nicotinamide, 1.25 mm N‐acetylcysteine, 12.5 ng mL^−1^ FGF‐7, 25 ng mL^−1^ FGF‐10, 500 nM A83‐01, 10 µm SB202190, and 10 µm Y‐27632.

### Biosensor Construction and Detection

All biosensor construction reagents were commercially sourced from ELISA kits. Coating antigen and blocking solution were sourced from the E‐UNEL‐H0025 Kit (Elabscience, China). Ab2‐HRP conjugate was obtained from E‐UNEL‐H0016 Kit(Elabscience, China). A detection solution, ultra‐sensitive ECL chemiluminescent substrate, was provided by Biosharp (BL520A, China). The construction protocol sequentially delivered coating antigen solution (20 min), followed by PBS washing (3 times, 1 min), and blocking solution (20 min). For detection procedures, samples were introduced first (20 min), followed by PBS washing(3 times, 1 min), Ab2‐HRP solution (20 min), PBS washing (3 times, 1 min), and finally pre‐mixed detection solution infusion (1 min). Prior to capturing, all internal light sources within the instrument were turned off to create a dark chamber. The chemiluminescent signals were acquired through the CCD imaging module under an exposure time of 200 ms. All capture operations were controlled by remote computer software with consistent capture parameters. For the medium within the same organoid culture chamber, three replicate units were simultaneously detected to measure CEA secretion levels. The image processing was then performed with ImageJ to calculate its intensity.

To validate coating efficiency, anti‐mouse IgG‐FITC secondary antibody labeling combined with confocal microscopy (Nikon, Japan) was employed. The final concentrations of constructing reagents were selected from an orthogonal experiment: 5× coating antigen concentration, 1× blocking solution, 1:1000 diluted Ab2‐HRP, and 1:2000 detection solution formulation.

### Immunostaining

For immunostaining, organoids were recovered from Matrigel and then fixed in 4% paraformaldehyde for 15 min. The fixed organoids were then washed with 1 × PBST 3 times. Subsequent permeabilization with 0.2% Triton X‐100 (15 min, RT) was followed by equivalent PBST washing and blocking in 10% goat serum (3 h, 37 °C). To assess the proliferation in the organoid, mouse anti‐Ki67 (Abcam, ab15580) was used as a primary antibody, while goat anti‐mouse IgG (Abcam, ab150115) was used as a secondary antibody. To identify the expression of organoid, rabbit anti‐EGFR (Abcam, ab52894) was used as a primary antibody, while goat anti‐rabbit IgG (Abcam, ab150077) was used as a secondary antibody. Both staining using primary antibodies and secondary antibodies were followed by washing with 1 × PBST 3 times. The nucleus was stained using the DAPI solution (C0065, Solarbio, China). The fluorescent photo was captured by the confocal microscope (Zesis, Germany), and image processing was performed with ImageJ.

### Cell Viability

The cell viability was tested by Calcein‐AM/PI cell viability/cytotoxicity assay kit (Beyotime, China) and Cell Counting‐Lite (Vazyme, China), respectively. By incubating the Calcein‐AM/PI buffer with organoids for 30 min, the live or dead cells can be observed under a fluorescent microscope. To evaluate the overall activity of the organoids, the organoids were digested into individual cells with TrypLE Express (Thermo Fisher, USA). Cells were transferred to a black plate at a count of 1000 per well and assayed using a multifunctional microplate reader (Biotek, USA).

### ASMO Setup

The ASMO system consists of a control module, chip module, and detection module. The control module uses a programmable logic controller (LinKong, China) with external software to manipulate other functional components: pumps, gas tanks, flow meters, and valves were used to control liquid and gas flows in the chip; temperature sensors, humidity sensors, and CO_2_ regulators were employed to control the external environment for chip culture. All liquid reservoirs and gas tanks were connected to the inlets and outlets of the chip via polyvinyl chloride (PVC) hoses, with the connection points sealed using sealing plugs. The hardware components were interconnected by wires for signal transmission. The system was supplied with an operating voltage of 24 V, and the CCD was supplied with an operating voltage of 12 V.

### Clinical Sample Collection and Ethical Statements

PDOs were established from tumor specimens that were collected from lung cancer patients at Peking Union Medical College Hospital. This study was approved by the Ethics Committee of Peking Union Medical College Hospital (No. ZS‐3176). Written informed consent was obtained from all participants. Detailed pathological information for each patient was provided in Tables S2 and S3 (Supporting Information).

### Finite Element Analysis

To simulate the concentrations achieved by the drug mixing structures, the fluidic layer built using SolidWorks was imported into the COMSOL Multiphysics version 6.1 simulation platform for analysis. A 3D model was used with the Transport of Diluted Species physics field, and the Laminar Flow physics field was selected for the simulation. The mesh was constructed with a standard element size. The concentrations at the two inlets were set to 0 and 10 mol m^−^^3^, respectively. For the Laminar Flow physics field, a no‐slip boundary condition was applied. The inlet velocities were set to 1 cm min^−1^, and the outlet was set to zero pressure. The resulting concentrations at the outlets were obtained from the simulation.

### Data Analysis

GraphPad Prism 10 (GraphPad Software Inc., USA) was used for data analysis. An ordinary one‐way ANOVA was used for multiple comparisons, while uncorrected Fisher's LSD was used to calculate the P‐value.

## Conflict of Interest

The authors declare no conflict of interest.

## Author Contributions

Y.Z., D.W. contributed equally to this work. Y.Z., D.W., Z.H., X.W., A.Z., R.Y., T.L., Z.Z., Y.L., N.L., Z.W., N.Z., and Z.W. conceived and designed the study. D.W., Z.H., X.W., A.Z., R.Y., Z.W., and T.L. performed the experiments and data curation. Z.H., X.W., and Z.W. conducted the formal analysis. Y.Z., Z.Z., T.L., Y.L., N.L., and N.Z. contributed to methodology development. Z.W. and N.Z. acquired funding and provided resources. Y.Z. wrote the original draft. All authors discussed the results and reviewed the manuscript.

## Supporting information



Supporting Information

## Data Availability

The data that support the findings of this study are available from the corresponding author upon reasonable request.;
