# Supplementary material for: Non‐Destructively Quantifying the Whole‐Course Growth and Drug‐Response of PDOs by an Automatic Microfluidic System Utilizing Chemiluminescence Detection
Source: Adv Sci (Weinh). 2025 Oct 24;13(2):e12951. doi: 10.1002/advs.202512951 (PMC12786359; doi:10.1002/advs.202512951)
Supplement: Supplementary file 1 — Supporting Information [file ADVS-13-e12951-s001.pdf]

Non-Destructively Quantifying the Whole-Course Growth and Drug-Response of PDOs by An Automatic Microfluidic System Utilizing Chemiluminescence Detection.

Yu Zhang [1#], Daoyun Wang[2#], Zhicheng Huang[2], Nan Zhang[3], Zhina Wang[3], Xin Wu[1], Anlan Zhang[1], Runzhi Yang[1], Tong Li[1], Zhibo Zheng[2], Yuxiao Lin[2], Naixin Liang[2], Zewen Wei[1*]*

1 Department of Biomedical Engineering, School of Medical Technology, Beijing Institute of Technology, Beijing 100081, China.

2 Department of Thoracic Surgery, Peking Union Medical College Hospital, Chinese Academy of Medical Sciences and Peking Union Medical College, Beijing 100730, China.

3 Department of Pulmonary and Critical Care Medicine 2, Emergency General Hospital, Beijing 100028, China.

# Y.Z., D.W. contributed equally to this work.

*Correspondence should be addressed to Naixin Liang (liangnaixin@pumch.cn) or Zewen Wei (weizewen@bit.edu.cn).

Supplementary Data S1

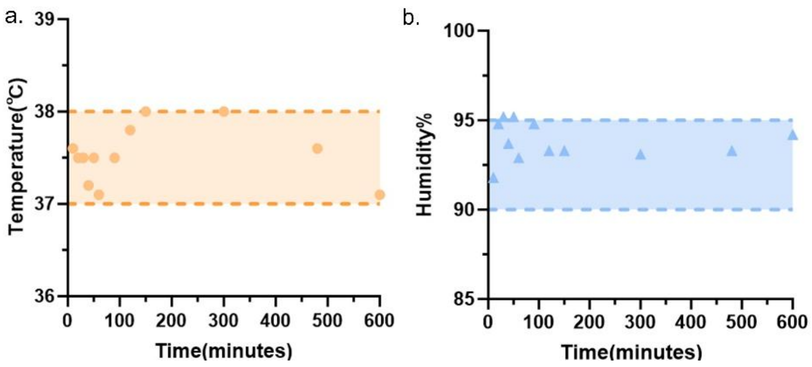

Figure S1, Stable environment inside the incubator controlled by self-regulating sensors. (a) The temperature inside the incubator was kept between 37 and 38°C during 10 hours. **(b)** The humidity inside the incubator was kept between 90% and 95% during 10 hours.

Supplementary Data S2

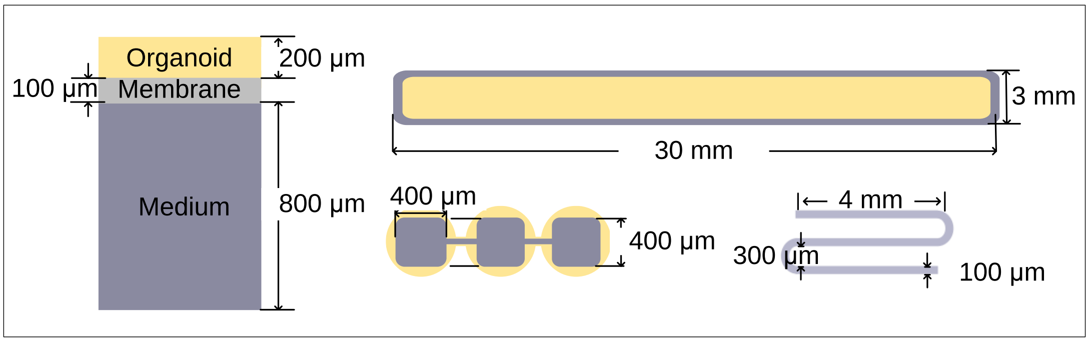

Figure S2, Key dimensions of CLOS-SEN chip. As shown in the cross-sectional view, the heights of channel for organoids (yellow), membrane (gray) and channel for medium (purple) were 200 μm, 100 μm and 800 μm, respectively.

From the top - down view, the parameters of the culture flow channel, detection flow channel, and drug generation flow channel were shown as follows: the main culture flow channel(purple) has a length of 30 mm and a width of 3 mm; the detection flow channel consists of three square - shaped detection units connected in a row, each square(purple) detection unit has a side length of 400 μm; the drug generation flow channel has a loop length of 4 mm, a channel space of 300 μm, and a channel width of 100 μm.

Supplementary Data S3

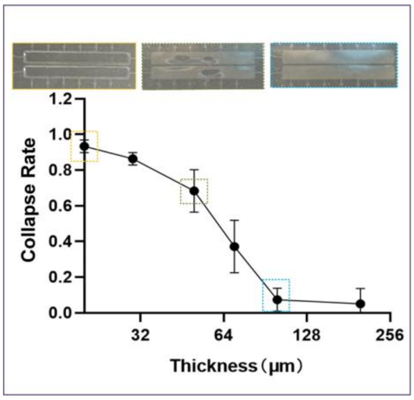

Figure S3, Design of layer thickness for CLOS-SEN chip. Relationship between layer thickness and collapse rate of film layer. The membrane layer that is too thin tends to collapse and too thick affects the elasticity of the membrane layer as a valve layer. A combined test of 100 μm was chosen as the final membrane layer thickness**.**

Supplementary Data S4

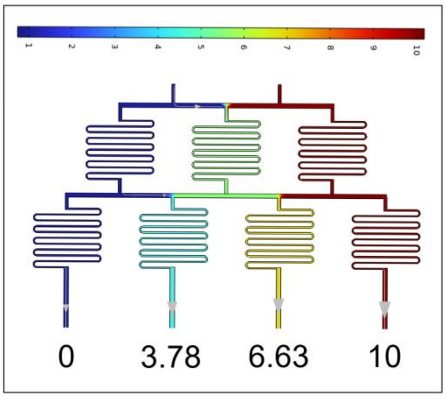

Figure S4, The FEA simulation of drug concentration generator. The values marked at the four outlets (0, 3.78, 6.63, and 10 μM) clearly showed the achievable range of drug concentrations, demonstrating the generator's capability to precisely control and produce multiple distinct concentration levels.

Supplementary Data S5

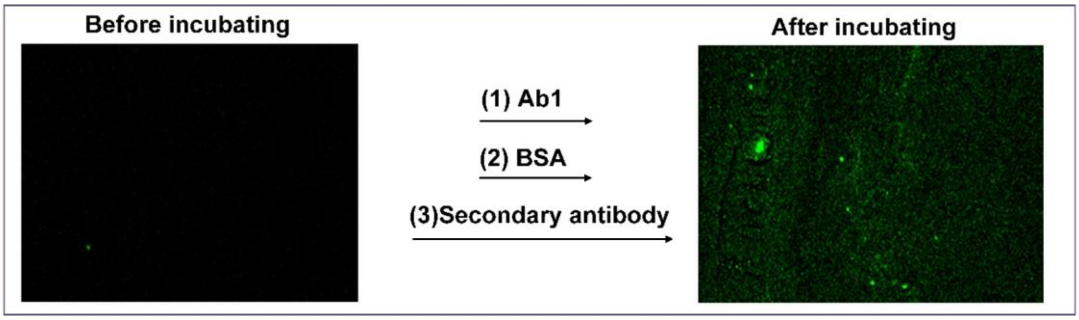

Figure S5, The immobilization of antibody on the PDMS. To confirm the successful antibody immobilization on PDMS, an immunofluorescence validation assay was performed. After blocking with 5% BSA, the PDMS substrate was incubated with a 488-labeled secondary antibody. Comparative confocal imaging analysis between pre- and post-incubation states demonstrated significantly enhanced fluorescence intensity, thereby verifying the successful antibody conjugation.

Supplementary Data S6

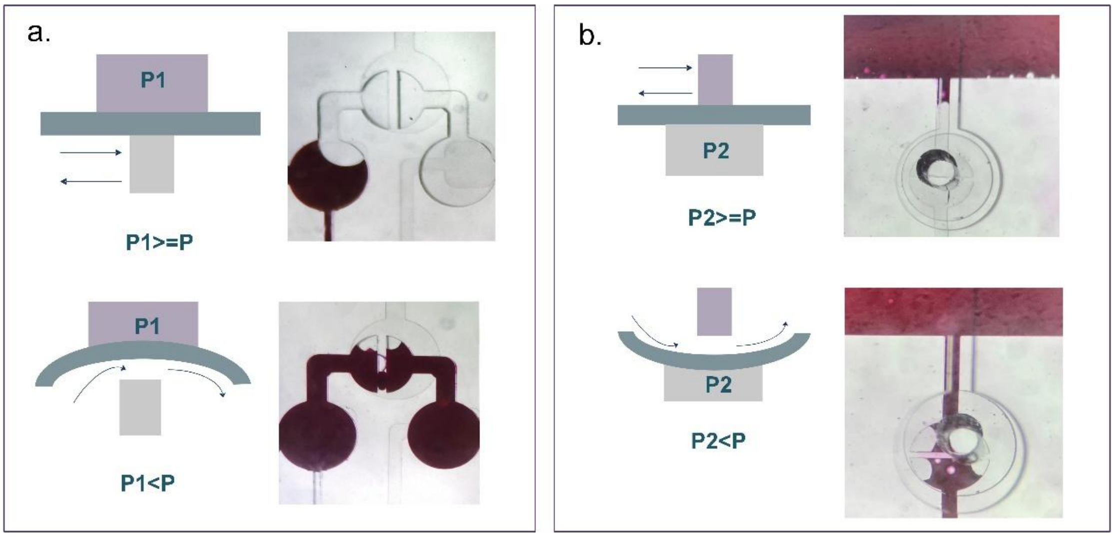

Figure S6, Multi-sequencing valves with temporal flow control for detection automation. (a) The valve was designed to control the fluidic connections among the different detection units by pneumatically actuating (compressing/relaxing) the membrane at specified timepoints. **(b)** The valve was designed to control the fluidic connections between the culture units and detection units by pneumatically actuating (compressing/relaxing) the membrane at specified timepoints.

Supplementary Data S7

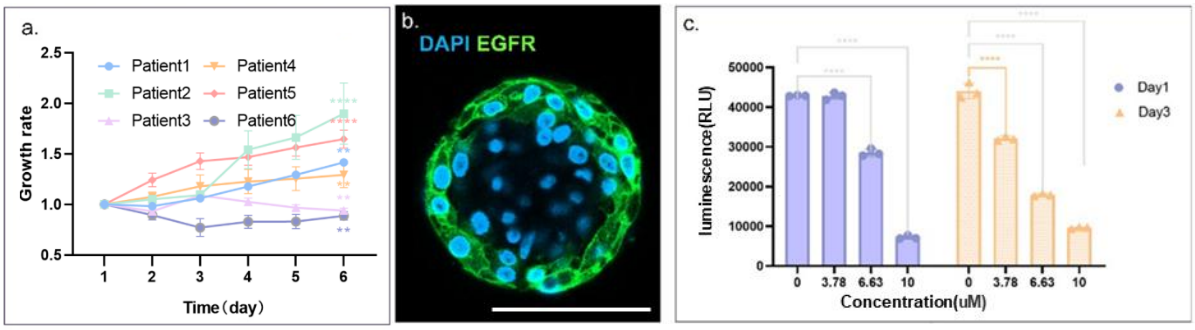

Figure S7, Conventional methods employed for PDO monitoring. (a) Continuous image tracking was performed for organoids derived from three patients maintained on the same focal plane. (n=6) Significant difference is based on the comparison of growth rates between Day 6 and Day 0. **(b)** Immunofluorescence staining of PDO. Scale bar: 50 μm. **(c)** ATP levels were quantified in PDOs from the same patient under four drug concentrations at two timepoints. T_1: Day 1(24 hours); T_2: Day 3(72 hours).

Supplementary Data S8

Sample number	Sample source	Concentration(ng/ml)	Minimum Detectable Liquid Volume for ELISA (μL)	Measured concentration by ELISA (ng/ml)	Minimum Detectable Liquid Volume for ASMO (μL)	Measured concentration by ASMO (ng/ml)	Significant	t-value	Adjusted P-value
1	Reference Standard	1.5	50	1.439	0.5	1.504	no	1.064	0.881433
2	Reference Standard	5		4.874		4.714	no	0.8379	0.907998
3	Reference Standard	10		9.976		9.551	no	2.544	0.326229
4	Used Culture Medium	unknown		3.437		3.374	no	0.268	0.977896
5	Used Culture Medium	unknown		0.9946		1.059	no	0.3857	0.977896
6	Used Culture Medium	unknown		1.797		1.833	no	0.2752	0.977896

Table S1, Comparison of CEA concentration detection using ELISA and the ASMO system.

Supplementary Data S9

Patient No.	Pathological Type	Sampling Method	Pathological Stage	TNM	Bulky/discrete lymph node metastasis	Treatment	EGFR mutation	Other Mutation	CEA-pretreatment	CEA-aftertreatment
1	AC	Surgery	IA2	T1bN0M0	No	R0 resection	EGFR E21 L858R +TP53	NA	1.6	1.3
2	SCC	EBUS-TBNA	IIIA	T3N2M0	Discrete	Albumin-Bound Paclitaxel + Carboplatin+Keytruda	NA	NA	NA	NA
3	AC	Surgery	IA3	T1cN0M0	No	R0 resection	EGFR E21 L858R	NA	2.3	1.4
4	AC	Surgery	IIIA	T3N2M0	Discrete	R0 resection	NA	CHEK1	9.4	3.1
5	SCC	Surgery	IIIA	T2bN2M0	Discrete	Neoadjuvant Therapy (Albumin-Bound Paclitaxel + Carboplatin+Keytruda) +R0 resection	NA	NA	7.2	4.9
6	AC	Surgery	IA2	T1bN0M0	No	R0 resection	NA	NA	1.6	1.3

***AC:** Adenocarcinoma

SCC: Squamous Cell Carcinoma

NA:NA indicates not applicable or undetectable, unable to detect.

R0 resection: The tumor lesion is completely removed, and there is no residual tumor cell at the cutting edge under the naked eye. Even under the microscope, no residual tumor components can be found at the cutting edge, achieving surgical cure effect.

Table S2, The detailed pathological information of patients.

Supplementary Data S10

Patient No.	Pathological type	Sampling Method	Pathological Stage	TNM	Bulky/discrete lymph node metastasis	EGFR mutation	Other Mutation	CEA pretreatment	CEA aftertreatment	Treatment before
3	AC	Surgery	IA3	T1cN0M0	No	EGFR E21 L858R	NA	2.3	1.4	In January 2025, after undergoing R0 resection with pathological stage IA3, the patient opted against postoperative adjuvant therapy.
7	AC	EBUS-TBNA	IIIB	T2N3M0	Yes	EGFR E19DEL+E20 T790 MC797S(Trans）+EGFR L718Q	NA	3	3.8	Jun 2015: Icotinib<br>May 2020: Acquired resistance → Switched to Aumolertinib<br>Apr 2025: Complex resistance → Started Sacituzumab tirumotecan
8	AC	Surgery	IIIA	T1bN2M0	No	EGFR E19DEL	NA	6.1	4.7	On July 1, 2025, the patient initiated adjuvant therapy with Osimertinib following surgical resection of EGFR-mutant non-small cell lung cancer.
9	AC	EBUS-TBNA	IV	T2N2M1	NA	EGFR E21 L858R	TP53、MET amplification	2	1.7	Feb 2022: Dacomitinib<br>Oct 2023: PD → Switched to Furmonertinib<br>Jul 2024: MET amplification detected → Started Osimertinib + Savolitinib<br>Dec 2024: AEs intolerable → Switched to Furmonertinib + Glumetinib
10	AC	EBUS-TBNA	IIIA	T2N2M0	Yes	NA	NF1+TP53	5.8	1.6	In August 2025, the patient received neoadjuvant chemoimmunotherapy with Pemetrexed (PEM), Carboplatin (CBDCA), and Pembrolizumab

Table S3, The detailed pathological information and disease journey of patients.

*__AC:__ Adenocarcinoma

SCC: Squamous Cell Carcinoma

NA:NA indicates not applicable or undetectable, unable to detect.
